# Applying the Guided Self‐Determination Counselling Method in Primary Healthcare Among People at Risk of Developing Type 2 Diabetes: A Qualitative Study

**DOI:** 10.1002/nop2.70346

**Published:** 2025-11-08

**Authors:** Beate‐Christin Hope Kolltveit, Elín Arnardóttir, Bjørg Frøysland Oftedal, Árún K. Sigurðardóttir

**Affiliations:** ^1^ Department of Health and Caring Sciences Western Norway University of Applied Sciences Bergen Norway; ^2^ School of Health, Business and Natural Science‐Faculty of Nursing University of Akureyri Akureyri Iceland; ^3^ Health Care Institution of North Iceland in Siglufjordur Siglufjordur Iceland; ^4^ Faculty of Health Sciences University of Stavanger Stavanger Norway; ^5^ Akureyri Hospital Akureyri Iceland

**Keywords:** guided self‐determination, nurses, primary healthcare, qualitative, risk of type 2 diabetes

## Abstract

**Aim:**

To explore how nurses experienced using the Guided Self‐Determination (GSD) counselling method in primary healthcare for the follow‐up of patients at risk of developing type 2 diabetes.

**Design:**

This study had a qualitative design.

**Methods:**

We used individual interviews with nurses in Norway (*n* = 5) and Iceland (*n* = 6) to collect data. The nurses had prior to this qualitative study participated in a randomised controlled trial, by applying the GSD counselling method in primary healthcare. The data was analysed by using qualitative content analysis.

**Results:**

The findings in this study resulted in the identification of three categories: (1) Promoting change in patient awareness and motivation, (2) Facilitation of health behaviour change, and (3) Challenges in applying the GSD method in patient consultations. The counselling method assisted the nurses to approach patients in a different way and to ask questions that stimulated personal reflection and increased the patients´ health awareness.

**Patient or Public Contribution:**

Patient representatives contributed in discussing the relevance of the study objectives and justification for undertaking the research, and in the development of adequate written patient information for the study.

## 1 Introduction

Type 2 diabetes (T2DM) is a significant global health challenge with long‐term complications, including heart disease, stroke, kidney failure and amputation (Alatorre et al. 2018). Findings from a retrospective population‐based cohort study emphasised that, regardless of genetic risk, being overweight, obese or having an inadequate lifestyle are key contributors to developing T2DM (Jakupovic et al. 2019; Ruze et al. 2023). It has been found that 38% of the global population is classified as overweight or obese (Koliaki et al. 2023). Thus, the prevention of risk factors might delay the onset of T2DM, as it has been found that, there is often a time lag of up to seven years between the onset of T2DM and its formal diagnosis (Kong et al. 2016).

Therefore, early detection of risk factors and lifestyle interventions is crucial to prevent further deterioration (Lemp et al. 2022). Behavioural interventions focusing on weight loss, diet, and physical activity are essential in preventing the development of T2DM (Hemmingsen et al. 2017; LeBlanc et al. 2018). A systematic review emphasised that the most effective interventions for achieving weight loss involve providing concise advice on diet and behaviour while encouraging behavioural change through regular follow‐up (Gostoli et al. 2024). Therefore, this study will focus on the follow‐up of patients at risk of developing T2DM in primary healthcare by applying the Guided Self‐Determination (GSD) counselling method.

## 2 Background

Primary healthcare plays a crucial role in identifying individuals at risk of developing T2DM and providing interventions to prevent disease progression (Haque et al. 2020). Nurses, as one of the primary points of contact in primary healthcare, are uniquely positioned to facilitate and support patients in adopting lifestyle modifications (Holloway et al. 2023). However, the follow‐up of people at risk of developing a chronic disease can be time‐ and resource‐ consuming, and primary care physicians (PCPs) report that there is limited time available for them to conduct this follow‐up (Moth et al. 2012; Svedahl et al. 2019). Globally, there are different approaches to organising primary healthcare. In Europe, traditional healthcare is generally provided in health‐care centres (nationally provided primary health care), where people can meet healthcare providers, such as nurses, physicians or physiotherapists. Those healthcare centres or clinics are the core of preventive health care and in supporting people to manage their illnesses (Meld. St. 19 (2014–2015)). In the primary healthcare clinics nurses often counsel and allocate care and independent treatment to patients frequently in collaboration with physicians.

In both Iceland and Norway, patients identified as at risk of developing T2DM constituted a novel patient group for nurses in primary healthcare settings. Although these two countries share many similarities, their primary healthcare systems are organised differently.

In Norway a PCP scheme was established in 2001 and this PCP scheme was supposed to guarantee all citizens the right to high‐quality primary care led by a specific PCP. In Norway the majority of people living with chronic diseases such as T2DM or who are at risk of developing T2DM are cared for by PCPs in primary healthcare (Helsedirektoratet (Norwegian Health Directory) 2018, 2020). Unlike other healthcare systems in Europe, the minority of PCPs in Norway employ registered nurses to take care of a broad range of work including counselling people with T2DM in diabetes management and healthy lifestyle or follow‐up of people at risk of developing T2DM. In Iceland, primary healthcare is financed by the state and primary healthcare clinics are scattered around the country, based on the distribution of settlements and challenging travel conditions in winter. In the primary healthcare clinics, physicians, nurses, physiotherapists and other healthcare professionals are employed (Sigurgeirsdóttir et al. 2014). In 2018 a Development Centre for Primary Healthcare in Iceland (DCPHI) on a national level was established. The DCPHI leads professional development within primary healthcare and works to coordinate procedures between professionals and supports physicians, nurses and other health care professionals by for example providing clinical guidelines through an active external and internal website (The Primary Healthcare of the Capital Area in Iceland 2024).

However, regardless of where nurses work, the core of their role in prediabetes and diabetes care is to promote and facilitate patient self‐management through education and support (Crowe et al. 2019; Fuentes‐Merlos et al. 2022). In a systematic review of 18 studies analysing nursing models of diabetes care, Crowe et al. (2019) found that nurse‐led interventions included the use of a patient‐centred approach and individualised education, which together led to confidence and independence among patients.

### 2.1 Theoretical Framework—The Guided Self‐Determination Counselling Method

There are many interventions designed to stimulate patients' motivation and empowerment. Motivational Interviewing (MI), Illness‐Integration Support (IIS), and Guided Self‐Determination (GSD) each offer distinct strengths depending on illness stage, patient needs, and clinical complexity. MI is valuable when patients express ambivalence; IIS is often suited to the early stages of T2DM and other chronic conditions; and GSD is particularly useful for problem‐solving (Zoffmann et al. 2016). While many interventions have proven effective, successful translation into practice requires selecting approaches suited to the clinical context and patient preferences—especially in resource‐limited settings. Person‐centred care also demands investment in training, opportunities for practice, and active leadership ownership to ensure sustainability. In this study, we chose GSD due to its demonstrated benefits among patients with diabetes (Linnet Olesen and Jørgensen 2023; Rorgemoen et al. 2021).

This counselling method is an evidence‐based and structured approach that facilitates patient‐centred care by fostering motivation, encouraging self‐reflection, and supporting the establishment of personal goals (Zoffmann et al. 2008; Zoffmann and Kirkevold 2012). GSD is rooted in research and theoretical frameworks related to empowerment, self‐determination, life skills, and humanistic principles. It serves as a counselling method designed to empower individuals living with chronic illnesses (Zoffmann and Kirkevold 2012). Moreover, a previous study has shown that the GSD method enhanced the nurses' counselling competence in consultations with people with type 2 diabetes (Oftedal et al. 2017). The primary aim of this method is to enhance patients' capacity to effectively manage the complexities of their conditions by equipping them with the necessary skills for proficient self‐management. The GSD method applies prefixed reflection sheets that serve as a supportive tool to promote autonomy by aiding individuals in setting goals for health promotion, deepening their understanding of personal values, identifying opportunities, and thus facilitating a readiness to change (Zoffmann et al. 2008). The reflection sheets were utilised in subsequent consultations with nurses, to address challenges, set goals, develop action plans, and explore other primary concerns that patients might have at that time. The GSD counselling method recognises that conflicts may arise due to an individual's life perspective, resistance to integrating ‘disease or risk symptoms’ into their daily routine, and the impact of these symptoms on their overall well‐being.

### 2.2 Aim

This qualitative study explored how nurses experienced using the GSD method in primary healthcare for the follow‐up of patients at risk of developing type 2 diabetes.

## 3 Methods/Methodology

### 3.1 Design

The study used a qualitative design with an inductive approach to explore the experiences of the nurses. The data were collected by means of semi‐structured interviews and analysed using qualitative content analysis (Graneheim and Lundman 2004).

### 3.2 Recruitment

We invited registered nurses in Norway and Iceland to participate in this study. These nurses were asked to participate due to their participation in a randomised controlled trial in Norway (*n* = 5) and Iceland (*n* = 6) where the GSD method was the intervention (Arnardóttir et al. 2024; Graue et al. 2023).

### 3.3 Participants

All the invited nurses (*n* = 11) accepted to participate. The nurses were all women in the age from 26 to 57 years. They had work experience as nurses from 5 to 33 years. Three of the nurses were diabetes nurse specialists.

### 3.4 Study Context and Use of the GSD Method

The randomised controlled trial was a nurse‐led follow‐up in primary healthcare to enhance patient centred care and facilitate lifestyle changes among individuals at risk of developing T2DM (Graue et al. 2024). The nurses in Norway worked in rural places and the population there was from 1000 to 15,000 inhabitants. The nurses in Iceland worked in rural places, in the three largest primary healthcare clinics in North Iceland, with a population from 3500 to 20,000 inhabitants (Arnardóttir et al. 2024).

Utilising the GSD counselling method, which incorporated semi‐structured reflection sheets, nurses applied a set of communication skills such as mirroring, active listening, and values clarification to help participants reflect on challenges related to their life and health with diabetes. This approach aimed to encourage and empower individuals to improve their self‐management of the condition.

To equip healthcare professionals with the necessary skills, the nurses had a comprehensive training program (Arnardóttir et al. 2024; Graue et al. 2024) that included:

1. An introductory presentation for the nurses, outlining the GSD counselling method and its application in the study.
2. Two workshops, a GSD reading list, and follow‐up supervised exercises.
3. Individual observation sessions with a certified GSD supervisor who is the first author.

### 3.5 Data Collection

We collected data by individually interviewing the participants using Zoom or telephone between January 2021 and June 2021 in Norway, and in May and June 2022 in Iceland. All interviews were conducted by the first author and the last author.

The interview guide was developed by the researchers based on the aim of the study and the literature (Linnet Olesen and Jørgensen 2023). The first author, a diabetes specialist nurse with extensive experience in primary healthcare and qualitative methods, initially drafted the guide, and then the co‐authors, all experienced qualitative researchers, provided feedback to refine and finalise the guide. The interview guide focused on topics such as nurses' experiences participating in the GSD program and applying the GSD counselling method in the follow‐up of individuals at risk of developing T2DM. The themes and research questions in the interview guide covered the following areas:

Experiences with the project:

- What are your overall experiences with participating in this project?
- Was there anything specific that you found to be negative or positive in the way you worked here?

Changes in work approach:

- How did you experience the follow‐up compared to what you are used to in your usual practice?
- What are your experiences using the GSD method? Was there something different about this way of working? How do you think the patients experienced this method?

Counselling:

- How did you find the experience of counselling patients in this project?
- How did you apply the GSD method in the follow‐up?
- Do you have any thoughts or suggestions for how counselling could be improved in primary healthcare?

Follow‐up questions like, ‘can you provide examples?’ or ‘could you elaborate further on your thoughts and reflections’ were asked to elucidate the participants' experiences.

The interviews lasted from 45 to 75 min.

### 3.6 Analysis

The interviews were transcribed verbatim and inductive content analysis following the methodological approach of Graneheim and Lundman (Graneheim and Lundman 2004) was applied. First, the authors read and reread the interviews, reflected on the data and discussed the analyses to be able to identify the manifest and latent content and increase the trustworthiness of the analysis (Elo et al. 2014). Meaning units based on words, sentences or paragraphs were selected. Then meaning units were condensed based on their content; the condensed meaning units were coded, At last categories were constructed as suggested by Graneheim and Lundman (2004). Then the other authors scrutinised the analyses and came up with minor suggestions for improvements.

We achieved enough information power (Malterud et al. 2016) to answer our research question after 11 interviews and the nurses did not report new and different perspectives and issues regarding their experiences of using the GSD counselling method. The sample can be said to be homogenous, as the inclusion criteria were to be a nurse and have experience in using the GSD counselling method. A systematic review has demonstrated that 9–17 individual interviews are sufficient to gain rich and valid data (Hennink and Kaiser 2022).

### 3.7 Ethical Considerations

The study has obtained ethical approval from the South‐Eastern Norway Regional Committee for Medical and Health Research Ethics (2019/28/REK sør‐øst A) and the Icelandic National Bioethics Committee (VSN) (VSN‐19‐080‐V1). The project was carried out in accordance with the Helsinki Declaration and reported in accordance with the COREQ (COnsolidated criteria for REporting Qualitative research) Checklist Appendix S1 (Tong et al. 2007). Further information can be obtained from ClinicalTrials.gov (ID: NCT04076384) and (ID: NCT04688359). The participants were informed that their participation was voluntary, and that they could withdraw from the study with no consequences. Anonymity was preserved by using numbers instead of names in all transcripts. The Western Norway University of Applied Sciences and School of Health Sciences University of Akureyri, Iceland are the responsible research institutions where we stored the data on a secure research server. Only the principal investigator and some clearly identified members of the project group had access to the data.

### 3.8 Rigour and Reflexivity

We enhanced the credibility of the study through our design of the study and the use of inductive qualitative content analysis guided by Graneheim and Lundman (2004). To enhance rigor, we discussed categories, codes and meaning units in the data several times from the whole data set, but also diving into parts of the data. This was done to analyze and interpret the underlying meaning within the data. The development of the semi‐structured interview guide helped us ensure that all relevant areas were explored consistently and allowed us to elaborate more when interesting information occurred. Dependability was upheld by following a systematic approach in data analysis, with the authors reading and re‐reading interview transcripts and discussing their interpretations to develop consensus. To enhance our transferability, we have explained the context in which the study took place and given information comprising the informants' years of experience as a nurse and specialist education and work location to help readers consider the study's applicability to similar settings. However, we have to acknowledge our preconceptions and the cultural and healthcare differences between Norway and Iceland, and we have tried to carefully consider these important issues in our interpretation of our findings.

## 4 Findings

In the analysis we identified three categories (*1) Promoting change in patient awareness and motivation, (2) Facilitation of health behaviour change and (3) Challenges in applying the GSD method in patient consultations*.

### 4.1 Promoting Change in Patient Awareness and Motivation

This category encapsulates the experiences of nurses indicating that the GSD method plays a significant role in enhancing patients' motivation and awareness. The nurses observed that the GSD method facilitated deeper and more meaningful dialogues with their patients. During initial consultations, they worked collaboratively with patients to identify challenges affecting their lives. Subsequent follow‐up sessions focused on addressing these challenges, which often related to difficulties in managing daily life and making healthy lifestyle choices. The GSD method encouraged nurses to ask reflective questions, fostering patients' personal insights and prompting them to explore strategies for healthier behaviour. Nurses emphasised the importance of allowing patients time for reflection, which helped them generate ideas for sustainable lifestyle changes.

Nurses reported a notable shift in patients' awareness and motivation during these consultations, characterised by a heightened understanding of their health condition and a stronger commitment to adopting healthier lifestyles. The use of the GSD method was experienced to empower patients to take an active role in managing their health. One nurse expressed this perspective:

The GSD method initiates an awareness among patients, so that in the long run it may well lead to better handling of their daily life for some. I saw that the GSD method facilitated that kind of awareness (Nurse 10 Norway)

Another said:

I think, maybe, at least five out of seven of the patients have shown success since they actually all expressed it themselves in the third consultation that this study just came at a good point in their lives and just like that changed their lifestyle (Nurse 1, Iceland).

Through implementing the GSD method, nurses gained communication tools that enhanced their patient‐centred approach. Rather than assuming the role of problem solvers, they embraced a counselling role, supporting patients in their individual needs. The GSD reflection sheets used during consultations facilitated this shift, enabling nurses to approach and motivate patients. Techniques such as mirroring and active listening were identified as particularly effective in promoting patient reflection and behaviour change. As one nurse articulated:

I made a conscious effort to employ mirroring techniques as much as possible, which helped uncover the underlying issues people were facing. As a result, individuals became more attentive to their health and more proactive in exploring ways to improve (Nurse 3, Iceland)

Nurses also gained deeper insights into the emotional and practical challenges faced by their patients. These challenges often surfaced early in the consultations, with patients showing a surprising willingness to share personal thoughts and struggles during the first meeting. In subsequent sessions, patients reflected further on these challenges and discussed them in greater detail, fostering a collaborative and supportive dialogue. One nurse said:

Three of the consultations were particularly challenging, as the patients faced numerous issues, including psychological difficulties. I was surprised by how openly they shared with me during the very first consultation, which was unexpected. However, the sessions progressed positively; during the subsequent meetings, they expressed feelings better, allowing us to continue discussing their concerns in greater depth (Nurse 5, Iceland)

While the nurses observed a positive shift in motivation among many patients, they also expressed frustration when encountering patients who appeared unmotivated to make lifestyle changes. This challenge was illustrated in the following quotation:

A woman came to the consultation, and you could see that she had already given up. She had at high risk for developing type 2 diabetes and had to do something, but she was not motivated to change health behaviour. When she came back for the last consultation, she told me that she hadn't done anything (Nurse 3, Iceland).

A similar sentiment from another nurse was expressed:

Some patients said that they had the attitude that if they were going to do something about their health, it had to be because they otherwise would drop dead from a heart disease or get a serious disease. I just had to realize that this was not something they were so motivated for; this was experienced a bit challenging to me (Nurse 3, Norway).

These experiences highlight the variability in patients' readiness to engage in lifestyle changes, underscoring the emotional and professional challenges faced by nurses in supporting individuals with differing levels of motivation.

### 4.2 Facilitation of Health Behaviour Change

Patients' motivation and awareness of prediabetes seemed to translate into active behavioural changes. Thus, this category reflects how the GSD method led to concrete behavioural changes in patients.

The nurses reported that the GSD method facilitated positive changes in patients' health behaviour by fostering self‐reflection and clarifying areas for improvement. The use of reflection sheets was experienced as important in guiding patients to focus on actionable changes to enhance their health. Unlike regular consultations, the GSD method provided a less time‐restricted environment, allowing space for deeper reflection and facilitating change. However, the nurses observed that behaviour change was a process, requiring time for patients to adapt and implement sustainable changes.

The nurses highlighted the importance of using communication techniques that emphasised self‐determination and personal goal‐setting. These strategies were effective in supporting patients to make meaningful adjustments to their diet and physical activity. Patients frequently expressed to the nurses that the consultations had equipped them with new skills and knowledge about healthy lifestyles. One nurse reflected:

Some patients said they were very pleased. Some didn't say anything. However, many said that they had learned a lot about diet and exercise, that they had become more aware of what they actually were doing (Nurse 3, Norway).

Another nurse noted the importance of self‐responsibility in the process:

In the first consultation … and when they came back to the second consultation, they referred to what I had said and stated, ‘Yes, you told me what I should do on my own'. So they experienced that it was they themselves who had to make an effort. They gained a better self‐understanding of what they themselves had to do (Nurse 4, Norway).

Nurses also observed that the issues discussed during the initial consultations often required time to mature before patients were ready to accept and act on them. As one nurse explained:

Then I noticed that things had to mature … many wanted their life to stay in the same way as it used to be. But I saw that the next time they came back, what we talked about last time had somehow matured (Nurse 5, Norway).

The structured yet flexible approach of the GSD method allowed for individualised care, which was viewed as central to health change behaviour. Reflection sheets helped patients uncover connections between life challenges and health behaviours. For instance, one nurse described how a patient, through the structured reflection process, realized that unresolved trauma was impacting her ability to focus on lifestyle changes:

Using unfinished sentences was interesting. Filling out the happiest moment of your life and the saddest moment of your life, it was interesting because every person said when their child was born and when they met their partner. And then when you hear what happened, the saddest thing, then you can realize that in some of the patients they had something, some trauma or challenges that was a part of the problem … we talked, and she told me the saddest part of her life is when she lost her brother in a car accident. … I asked her, ‘Do you think you have PTSD?’ … She was like, ‘Yeah maybe,’ and afterwards, she has been to a psychiatrist, and now she has been in therapy and diagnosed with PTSD. … It was amazing to hear that. Like she has been realizing that she has been dealing with so much in life that she is not coping with. … Then we discussed, like, you cannot just start eating healthy and going to the gym if that is your goal. There is so much more you have to deal with first (Nurse 6, Iceland).

The GSD method encouraged nurses to ask patients challenging questions that provoked reflection, which was essential for guiding them toward improved health change. Although some patients chose not to answer directly, nurses observed that these questions often stimulated individual reflection, which later facilitated behaviour change. One nurse emphasised:

You have to dare to ask difficult questions … the patient can choose not to answer, that is fine because you have stimulated them to reflect (Nurse 1, Norway).

### 4.3 Challenges in Applying the GSD Method in Patient Consultations

This category comprises the complexity and challenges in applying the Guided Self‐Determination method in the follow‐up, even though nurses reported that it facilitated the dialogue between the patients and nurses. Across the data, Icelandic nurses generally described the method in more positive terms than the Norwegian nurses. For example, one Icelandic nurse stated: ‘It was really good. I don't know what I would have done without it. It is good to use. I get to know the patients more in depth and what their challenges are’. The Norwegian nurses tended to express more reserved evaluations of the method. As one Norwegian nurse noted: ‘The method is useful for some patients, but it is difficult to apply’. However, nurses from both countries perceived the use of the GSD method in patient consultations as demanding. This method enabled consultations to centre more profoundly on individual patients and their unique life circumstances. Nurses reported that, while the GSD method required additional effort, it facilitated a deeper understanding of their patients. The structured framework of the method supported the exploration of challenges and priorities unique to each individual. One nurse remarked:

There has been a lot of extra work with this, but at the same time it has been good to know that you are getting better contact with the patient (Nurse 2, Norway).

Another nurse highlighted the benefits of this approach, stating:

After all, I found it positive to get to know the patients better … It has been beneficial to get a little under the skin of the patient, understand how different things affect the situation (Nurse 1, Norway).

A critical aspect of the GSD method was providing patients with the time and space needed to reflect on their lives, which often led to emotional expression during consultations. Nurses viewed this as a valuable development, although it also presented challenges for both patients and nurses. The nurses experienced that some patients faced emotional difficulties during the consultations, with one nurse sharing an example:

It was interesting, and it was difficult as well, but it was good to just ask the patients, ‘What do you think you have to do?’ To make them reflect, I think that was good. And active listening as a way of communicating I think also was super important. Because you felt … some people were starting crying, some people had feelings they were not used to talking about when they came to the healthcare clinic. So, I feel some of them had a really hard time after the consultation. I have one patient that I think she felt she lost a little bit of her emotions during the consultation, and she didn't want to show those emotions (Nurse 3, Iceland).

The use of reflection sheets, a key component of the GSD method, was identified as both advantageous and challenging. While some nurses experienced that the patients perceived the repetitive nature of these tools as burdensome, others found that this repetition encouraged them to engage more deeply with their struggles. One nurse noted:

…there is a lot of repetition in this so it makes people open up and go into themselves a little and look at their difficulties or problems (Nurse 4, Iceland).

Despite the method's benefits, its integration into existing clinical workflows posed some challenges for the nurses, particularly due to the additional time and resources required. Moreover, nurses reported that some patients, especially older individuals, found the reflective and patient‐centered approach unfamiliar. Older patients often expected direct advice rather than participatory dialogue. One nurse articulated this challenge:

You could see people were like, ‘Am I just coming here, I can talk about myself and you're just going to listen?’ That was really positive, but it was as well really difficult especially with older patients; they had a really hard time to think for themselves because they are used to being told, ‘You are supposed to do that, you're supposed to do that.’ Some of them asked me, ‘Can you just please tell me what I am supposed to do? Can you just give me some pointers? Something on paper so I could do it?’ It was hard to get them to understand they need to find ways themselves also (Nurse 6, Iceland).

Working with older patients using the GSD method presented unique difficulties, as many of these individuals did not perceive the need for lifestyle changes, often attributing this to their age. One nurse reflected on this issue:

I feel I was able to challenge the patients to do something to change their lifestyle, but I experienced it a bit difficult with older patients. Some of them said, ‘I am very healthy, there is nothing wrong with me and there is nothing I want to change.’ I talked a bit about nutrition, but I didn't tell them what to do (Nurse 6, Iceland).

Overall, the GSD method was found to facilitate meaningful and reflective dialogues between nurses and patients, yet its implementation posed significant challenges. While the approach helped build rapport and foster deeper understanding, it also demanded additional time, effort, and emotional labour from healthcare providers. The difficulties were particularly pronounced when working with older patients, who were less familiar with the reflective, self‐determined approach.

## 5 Discussion

The findings from this study demonstrate that the nurses experienced that the GSD counselling method has significant potential in enhancing patient‐centred care and supporting behaviour changes for patients at risk of developing T2DM within primary healthcare settings. Still, despite its promise, the study also highlights challenges in implementing the GSD approach effectively in primary healthcare clinics both in Norway and Iceland.

A key finding in this study is the shift in patient awareness and motivation, a result that aligns closely with existing literature on the importance of patient‐centred approaches in diabetes care (Gostoli et al. 2024; LeBlanc et al. 2018). Motivation is a psychological drive that initiates, guides, and sustains goal‐oriented actions (Ryan et al. 2022). This emphasises motivation as a dynamic force that not only triggers action but also maintains it over time, guiding individuals toward achieving specific goals (Ryan 2017). Nurses reported that patients often did not fully comprehend the risk factors associated with prediabetes, even when they were explicitly informed and some came to the consultation without any motivation to change their lifestyle. This reflects a common issue in diabetes management, where patients may receive information but lack the depth of understanding and motivation necessary to prompt lifestyle changes (Dambha‐Miller et al. 2016). However, once engaged with the GSD process, patients became more aware of their health risks, which motivated them to adopt healthier behaviours. This shift highlights the power of structured methods, such as GSD, in raising awareness and fostering patient responsibility for their health. By encouraging self‐reflection and goal setting, the GSD method indicates that patients might be empowered to actively participate in managing their risk factors for T2DM. This finding is particularly crucial given the global burden of T2DM and the growing need for preventive measures that focus on early detection and lifestyle modification. The fact that nurses noted this increase in patient awareness suggests that structured interventions like GSD could be integral to preventing the onset of T2DM through promoting a more proactive approach to health. Applying GSD in a primary care setting within people at risk of developing T2DM is to our knowledge not usual; however, it is not less important due to the reduction of early onset of a chronic and costly disease.

Another important finding is the facilitation of change in health behaviour. This finding highlights the usefulness of the GSD method in promoting healthier lifestyle practices among patients. Behavioural interventions, particularly those focused on weight reduction, dietary modification, and increased physical activity, are widely recognised as essential strategies in the prevention of T2DM (French et al. 2023); however there is also a need for optimising behavioural interventions in the future.

Through the GSD method, patients were able to identify personal barriers to lifestyle changes and develop practical strategies for overcoming these challenges. This self‐reflective approach, enhanced by ongoing support from nurses, empowered patients to set achievable health goals and make progressive steps toward a healthier lifestyle. Such an approach aligns with literature emphasising that self‐awareness and goal setting are central components of effective behaviour modification in chronic disease prevention (Novak et al. 2013). Effective management of risk factors or prediabetic conditions presents a complex and multifaceted challenge, necessitating comprehensive knowledge, adequate support, and robust resources. This process requires not only the commitment and involvement of patients but also coordinated, collaborative efforts among healthcare providers to ensure optimal outcomes. Nevertheless, the findings indicate that the GSD method does not suit everyone. Some participants, particularly older individuals, expressed that they neither saw the need for change nor had the motivation to make health‐related changes in their lives. Among older individuals, motivation may not even be evident, as the potential gain can appear uncertain. This does not imply that the GSD method is ineffective; quite the opposite, it can help identify patients who are unmotivated, enabling nurses to avoid investing significant time in those who explicitly express, through the reflection sheets, that they lack motivation for health behaviour change. This allows nurses to focus their time and efforts on patients who are motivated and willing to engage in the necessary actions to prevent type 2 diabetes and improve their health behaviour.

The role of nurses in facilitating these behavioural changes is particularly significant. In Iceland especially, but also increasingly in Norway the nurses are primary points of contact within the healthcare system and manage a broad range of responsibilities in primary care (Helsedirektoratet (Norwegian Health Directory) 2018, 2020; Sigurgeirsdóttir et al. 2014). Their involvement in patient counselling and lifestyle interventions underscores their critical role in risky behaviour management and chronic disease prevention. By employing the GSD method, nurses were equipped with a structured framework that incorporated active listening, reflective mirroring, and values clarification, thereby fostering more personalised and collaborative interactions with patients. This approach not only enhanced communication but also made the behaviour change more patient‐centred and adaptable to individual needs, as supported by another study (Kolltveit et al. 2023; Oftedal et al. 2017). These findings underscore the value of structured, nurse‐led interventions in promoting sustainable lifestyle changes and the potential of the GSD method as a model for chronic disease prevention efforts in primary care settings. Future research could investigate the applicability of the GSD method across diverse healthcare settings and examine its long‐term effects on patient health outcomes in the prevention of type 2 diabetes mellitus.

While the GSD method offers significant benefits, its integration into routine clinical practice has presented notable challenges, as outlined in our findings. One primary challenge involved the time and resource constraints prevalent in primary care settings, a limitation that aligns with findings from other studies on the implementation of complex interventions in similar environments (Kolltveit et al. 2023). Nurses in both Norway and Iceland reported that, although the GSD method facilitated deeper and more meaningful patient interactions, it also demanded considerable additional time and effort. This added burden may pose a barrier within primary care systems that are often already overstretched.

Furthermore, a nuance was observed between the Norwegian and Icelandic nurses in how they described the method. The Icelandic nurses generally appeared more positive and open to adopting the GSD method than the Norwegian nurses, who tended to express a more reserved stance. Given the limited sample size we can only speculate about the reasons for this difference. Possible explanations include personal and cultural factors, as well as systemic differences in primary care organisation and funding between the two countries (Iversen et al. 2016). In Iceland primary healthcare is often delivered through an integrated care model that includes group practices comprising nurses, physicians and other healthcare professionals (Developmentcenter for primary healthcare on Iceland‐Utviklingssenter for primærhelsetjenester på Island 2024).

These variations suggest that the successful implementation of the GSD method may be contingent upon the specific healthcare context, particularly the support structures available to nurses and the emphasis on preventive care within healthcare policies. Such findings indicate that while the GSD method has substantial potential, its effectiveness may be significantly influenced by the unique characteristics and resources of the healthcare setting.

## 6 Methodological Considerations

Our study was conducted in primary care settings in rural places in Norway and Iceland. Our findings cannot be extrapolated to other settings; however our findings might provide us with some more insights on how to apply GSD in some primary care settings. We interviewed all participants who could elaborate on the topic and thus help us gain a deeper understanding of applying GSD in primary care among nurses. However, a potential limitation is that participants may have been especially motivated to use GSD simply because they chose to take part in this study. It can be assumed that if this method had been implemented in a primary care setting without nurses' involvement in the decision‐making process, it might have led to the identification of different attributes than those reported by the participants.

The study utilised in‐depth interviews conducted via Zoom or telephone, which offered several advantages, including flexibility for participants and researchers, as well as the ability to engage participants across geographical locations. However, this approach may have influenced the richness of the data compared to face‐to‐face interactions. Non‐verbal cues, which can add depth to qualitative insights, were limited in telephone interviews and less pronounced in virtual meetings. Another notable limitation of the study was the absence of male participants, which may have restricted the diversity of insights and hindered a fuller understanding of the phenomenon being investigated.

## 7 Conclusion and Implication for Practice

The findings from this study have some implications for primary healthcare practice, particularly in the management of patients at risk of developing T2DM. The positive key aspects of the GSD method in promoting patient‐centred care and facilitating behavioural change indicate that this approach could be a valuable tool in preventive health. However, for its successful implementation, healthcare systems must address the challenges identified in this study, particularly the time and resource demands placed on nurses.

Given the growing burden of T2DM and the critical role of early intervention, integrating GSD or similar patient‐centred approaches into primary care could be a key strategy in reducing the incidence of T2DM. However, this will require adequate training for healthcare professionals, streamlined workflows to accommodate more in‐depth consultations, and the necessary structural support to ensure that nurses are not overwhelmed by the additional demands of implementing such methods.

In conclusion, the GSD method represents a promising approach for enhancing patient‐centred care and promoting behaviour change among individuals at risk of developing T2DM. It fosters increased patient awareness and motivation, facilitates meaningful health behaviour changes, and empowers nurses to take an active role in preventive care. However, challenges related to the method's integration into routine practice, particularly in terms of time and resource constraints, must be addressed. Tailoring the implementation of GSD to the specific healthcare context and providing sufficient support to healthcare professionals will be crucial for its long‐term success in preventing T2DM.

### 7.1 Patient and Public Involvement

A patient representative in Norway contributed to discussing the relevance of the study objectives and justification for undertaking the research. In addition, the user representative has contributed to decisions on methods related to data collection. The user representative participated in the development of adequate written patient information for the study. In Iceland, the patient representative contributed to discussing the relevance of the study objectives and justification for undertaking the research.

## Author Contributions

B.‐C.H.K. and Á.K.S. applied for funding for this study. B.‐C.H.K. and Á.K.S. designed the study with the involvement of B.F.O. B.‐C.H.K., Á.K.S. and E.A. collected the data. B.‐C.H.K., Á.K.S. and B.F.O. contributed to data analysis. B.‐C.H.K., Á.K.S., B.F.O. and E.A. contributed to drafting the manuscript and read and approved the final manuscript.

## Ethics Statement

The study has obtained ethical approval from the South‐Eastern Norway Regional Committee for Medical and Health Research Ethics (2019/28/REK sør‐øst A) and the Icelandic National Bioethics Committee (VSN) (VSN‐19‐080‐V1). The project was carried out in accordance with the Helsinki Declaration and reported in accordance with the COREQ (COnsolidated criteria for REporting Qualitative research) Checklist Appendix S1 (Tong et al. 2007). Further information can be obtained from ClinicalTrials.gov (ID: NCT04076384) and (ID: NCT04688359). The participants were informed that their participation was voluntary, and that they could withdraw from the study with no consequences. Anonymity was preserved by using numbers instead of names in all transcripts. The Western Norway University of Applied Sciences and School of Health Sciences University of Akureyri, Iceland are the responsible research institutions where we stored the data on a secure research server. Only the principal investigator and some clearly identified members of the project group had access to the data.

## Consent

All patients that participated filled out an informed consent form prior to participation.

## Conflicts of Interest

The authors declare no conflicts of interest.

## Supporting information


**Appendix S1:** nop270346‐sup‐0001‐AppendixS1.pdf.

## Data Availability

The data that support the findings of this study are available on request from the corresponding author. The data are not publicly available due to privacy or ethical restrictions.
